# Modelling and advanced characterization of framework materials

**DOI:** 10.1038/s42004-023-01071-5

**Published:** 2023-12-18

**Authors:** François-Xavier Coudert, Claire L. Hobday, Satoshi Horike, Monique A. van der Veen

**Affiliations:** 1 Chimie ParisTech, PSL University, CNRS, Institut de Recherche de Chimie Paris, Paris, France; 2 Centre for Science at Extreme Conditions and EaStCHEM School of Chemistry, The University of Edinburgh, King’s Buildings, David Brewster Road, Edinburgh, EH9 3FJ UK; 3 Department of Chemistry, Graduate School of Science, Kyoto University, Kitashirakawa-Oiwakecho, Sakyo-ku, Kyoto 606-8502 Japan; 4 Catalysis Engineering, Department of Chemical Engineering, Delft University of Technology, Delft, 2628 The Netherlands

**Keywords:** Metal-organic frameworks, Porous materials, Computational chemistry

## Abstract

*Communications Chemistry* is delighted to introduce a Collection of research works focused on the modelling and advanced characterization of framework materials. Here, the Guest Editors outline the themes within and look towards the future of the field.

The emergence of novel families of framework materials, including metal–organic frameworks (MOFs) and covalent organic frameworks (COFs), has been an important development in the field of nanoporous materials over the last 2 decades, with such frameworks displaying promise for a broad range of applications such as gas separation and storage, catalysis, energy storage, etc. As the number and diversity of known framework materials have skyrocketed, so have ongoing challenges in the understanding of their structure as well as their physical and chemical properties from a spatiotemporal viewpoint. To address this, the characterization and modelling of these materials have become a focal point in materials science research, with methodological developments of importance in advancing the field. In support of ongoing efforts in these directions, we are delighted to showcase in this Collection a range of research works that highlight advances and challenges at the frontiers of this exciting field. These papers cover three main themes: (1) flexibility, dynamics, and elasticity, (2) materials with a disorder or low crystallinity, and (3) establishing structure–property relationships.

## Flexibility, dynamics, and elasticity

Due to the nature of their constitutive interactions, and the high number of degrees of freedom, framework materials are intrinsically dynamic. Moreover, they often exhibit a strong response to changes in their environmental conditions, i.e., display a response to external stimuli in both their microscopic structure and properties. The theme of structural flexibility, dynamics, and elasticity has therefore been a major research area in this field, trying to understand the link between microscopic features and macroscopic behavior. The papers in this section illustrate the current research in this area, looking into the impact of phenomena such as linker rotation, local functional group dynamics, mechanical and thermal responses, structural phase transitions, and the coupling between flexibility and adsorption.

## Materials with disorder or low crystallinity

The last few years have seen a rapidly increasing number of framework materials that display disorder or low crystallinity—a trend expected to continue in the next decade. For these systems, characterization is key but rendered much more complex, both experimentally and computationally, than for cases of averaged/uniform crystalline systems. The research works featured in this section of the Collection focus on unraveling the structure of framework materials with heterogeneity in compositions and a wide range of structural periodicity. Advanced characterizations for molecular frameworks provide novel insight: the use of three-dimensional electron diffraction to better understand the structure, and genetic algorithms to rationalize and optimize synthesis conditions, for example. Moreover, defect-induced tuning of adsorption highlights the practical implications of understanding disorder in these materials.

## Structure–property relationships

Understanding the relationships between the structures and properties of materials is crucial for tailoring their performance, something which is particularly challenging in framework materials due to their multi-scale intricate structures and dynamics, as well as their large number of potential applications. Moreover, the complexity of the structural information obtainable has increased over time as the experimental and computational methodologies available push the boundaries of characterization. The papers highlighted here delve into the establishment of structure–property relationships for metal–organic frameworks and related materials, with a focus ranging from catalysis to gas adsorption. They use advanced synchrotron X-ray diffraction, in situ adsorption analysis, computational techniques, and machine learning to predict and optimize the properties of framework materials.

## Outlook

Reflecting on the commonalities of the above works on the characterization and modelling of framework materials, and the frontiers that they delineate, it becomes clear that these materials are far from being fully understood. The themes of flexibility, disorder, and structure–property relationships interconnect in forming a holistic picture of the intriguing world of framework materials. We see also that the development of a large number of techniques, both experimental and computational, is rendered necessary in order to fully understand these complex systems. Advances in fundamental understanding are, in turn, necessary for their use in practical and industrial applications.

We hope that this Collection of research works will inspire more collaboration and exploration, driving the field of framework materials towards new horizons and transformative applications.

